# Switch of the interactions between the ribosomal stalk and EF1A in the GTP- and GDP-bound conformations

**DOI:** 10.1038/s41598-019-51266-x

**Published:** 2019-10-14

**Authors:** Kei Maruyama, Hirotatsu Imai, Momoko Kawamura, Sonoko Ishino, Yoshizumi Ishino, Kosuke Ito, Toshio Uchiumi

**Affiliations:** 1 Department of Biology, Faculty of Science, Niigata University, Ikarashi 2-8050, Niigata, 950-2181 Japan; 2 Department of Bioscience and Biotechnology, Graduate School of Bioresource and Bioenvironmental Sciences, Kyushu University, Fukuoka, 819-0395 Japan

**Keywords:** X-ray crystallography, Ribosome

## Abstract

Translation elongation factor EF1A delivers aminoacyl-tRNA to the ribosome in a GTP-bound form, and is released from the ribosome in a GDP-bound form. This association/dissociation cycle proceeds efficiently via a marked conformational change in EF1A. EF1A function is dependent on the ribosomal “stalk” protein of the ribosomal large subunit, although the precise mechanism of action of the stalk on EF1A remains unclear. Here, we clarify the binding mode of archaeal stalk aP1 to GTP-bound aEF1A associated with aPelota. Intriguingly, the C-terminal domain (CTD) of aP1 binds to aEF1A•GTP with a similar affinity to aEF1A•GDP. We have also determined the crystal structure of the aP1-CTD•aEF1A•GTP•aPelota complex at 3.0 Å resolution. The structure shows that aP1-CTD binds to a space between domains 1 and 3 of aEF1A. Biochemical analyses show that this binding is crucial for protein synthesis. Comparison of the structures of aP1-CTD•aEF1A•GTP and aP1-CTD•aEF1A•GDP demonstrates that the binding mode of aP1 changes markedly upon a conformational switch between the GTP- and GDP-bound forms of aEF1A. Taking into account biochemical data, we infer that aP1 employs its structural flexibility to bind to aEF1A before and after GTP hydrolysis for efficient protein synthesis.

## Introduction

Protein synthesis on the ribosome is regulated by the action of several translational GTPase factors on the ribosome with their action being coupled to GTP hydrolysis^1–3^. The archaeal GTPase factor aEF1A (eEF1A in eukaryotes, EF-Tu in bacteria) delivers aminoacyl-tRNA to the ribosome in its GTP-bound form^4^. After codon recognition by the aminoacyl-tRNA and GTP hydrolysis, aEF1A is then released from the ribosome in its GDP-bound form. aEF1A also delivers aRF1 and aPelota in translation termination and mRNA surveillance pathways, respectively^5^. Therefore, the progress of the ribosome•aEF1A association/dissociation cycles mediated by GTP hydrolysis is related to efficient and accurate translation^3^.

The ribosomal protein called the “stalk” which is in the ribosomal large subunit plays a crucial role in efficient action of translational GTPases^3,6–8^. In archaea, the ribosomal stalk protein, aP1, and its anchor protein aP0 form a heptameric complex aP0•(aP1)_2_•(aP1)_2_•(aP1)_2_^9,10^. Our biochemical studies have indicated that the flexible C-terminal end region of aP1 (aP1-CTD) directly binds to both the GTP- and GDP-bound forms of aEF1A^11,12^, and suggest that it plays important roles in activity, including in recruitment of aEF1A•GTP to the ribosomal factor binding center and in activation of GTP hydrolysis^12,13^. In a previous study, we solved the crystal structure of the complex between aP1 and the GDP-bound form of aEF1A^12^. The structure showed that aP1-CTD forms an α-helix in the complex and interacts with a hydrophobic space between domains 1 and 3 of aEF1A•GDP. Comparison of the structures of the GDP- and GTP-bound conformations of aEF1A demonstrates a difference in the location of domain 1 relative to domains 2 and 3. Such a change is also observed for bacterial EF-Tu (these GDP- and GTP-bound conformations of EF1A/EF-Tu are sometimes referred to as the open and closed conformations, respectively)^14–18^. In the GTP-bound form of aEF1A (closed conformation), the space between domains 1 and 3, to which aP1-CTD binds in aP1•aEF1A•GDP (open conformation), is completely disrupted. Therefore, the stalk is expected to bind to aEF1A•GTP in a completely different mode to that when it is bound to aEF1A•GDP. However, no data is available on the structure of the aP1•aEF1A•GTP complex.

Elucidation of the molecular mechanism of binding between the stalk and aEF1A•GTP is important, because it would provide evidence for the action of the ribosomal stalk in recruitment to the ribosome of aEF1A•GTP associated with ligands (including aminoacyl-tRNA, aRF1, and aPelota), and also in the coupled GTP hydrolysis. Using archaeal samples, we here describe X-ray crystal structural analysis of the complex of aP1•aEF1A•GTP. We use a 17-mer peptide as an aP1-CTD sample and aPelota as a ligand of aEF1A to stabilize the GTP-bound state, and have determined the complex structure at 3.0 Å resolution. The structure shows that aP1-CTD binds to a hydrophobic cleft between domains 1 and 3 formed uniquely in the GTP-bound form of aEF1A. Moreover, the interactions between aP1-CTD and aEF1A•GTP seen in the crystal structure are evaluated by biochemical analysis using aEF1A mutants. The results suggest that, despite the large conformational change of aEF1A before and after GTP hydrolysis, interaction between aP1-CTD and aEF1A is maintained by switching of binding modes.

## Results

### Binding of aP1 to GTP-bound form of aEF1A

The ribosomal stalk protein aP1 promotes translation by recruiting aEF1A•GTP associated with its ligand (aminoacyl-tRNA, aRF1, or aPelota) to the ribosome. Therefore, we have analyzed the binding between aP1 and aEF1A•GTP in the presence of one of its ligands, aPelota. We initially attempted to prepare *Pyrococcus horikoshii* aPelota (_Pho_Pelota), because we were able to show binding of _Pho_P1 to the GDP-bound form of elongation factor aEF1A using *P*. *horikoshii*_Pho_EF1A and elucidated the crystal structure of the complex^12^. Unfortunately, *E*. *coli* expression of _Pho_Pelota was poor. However, we found that *Pyrococcus furiosus* aPelota (_Pfu_Pelota) was expressed at a sufficiently high enough level for the analysis. Furthermore, there is high homology in amino acid sequences between _Pho_Pelota and _Pfu_Pelota (Fig. S1). Therefore, we used _Pfu_Pelota with _Pho_P1 and _Pho_EF1A for further biochemical binding assays.

**Figure 1:**
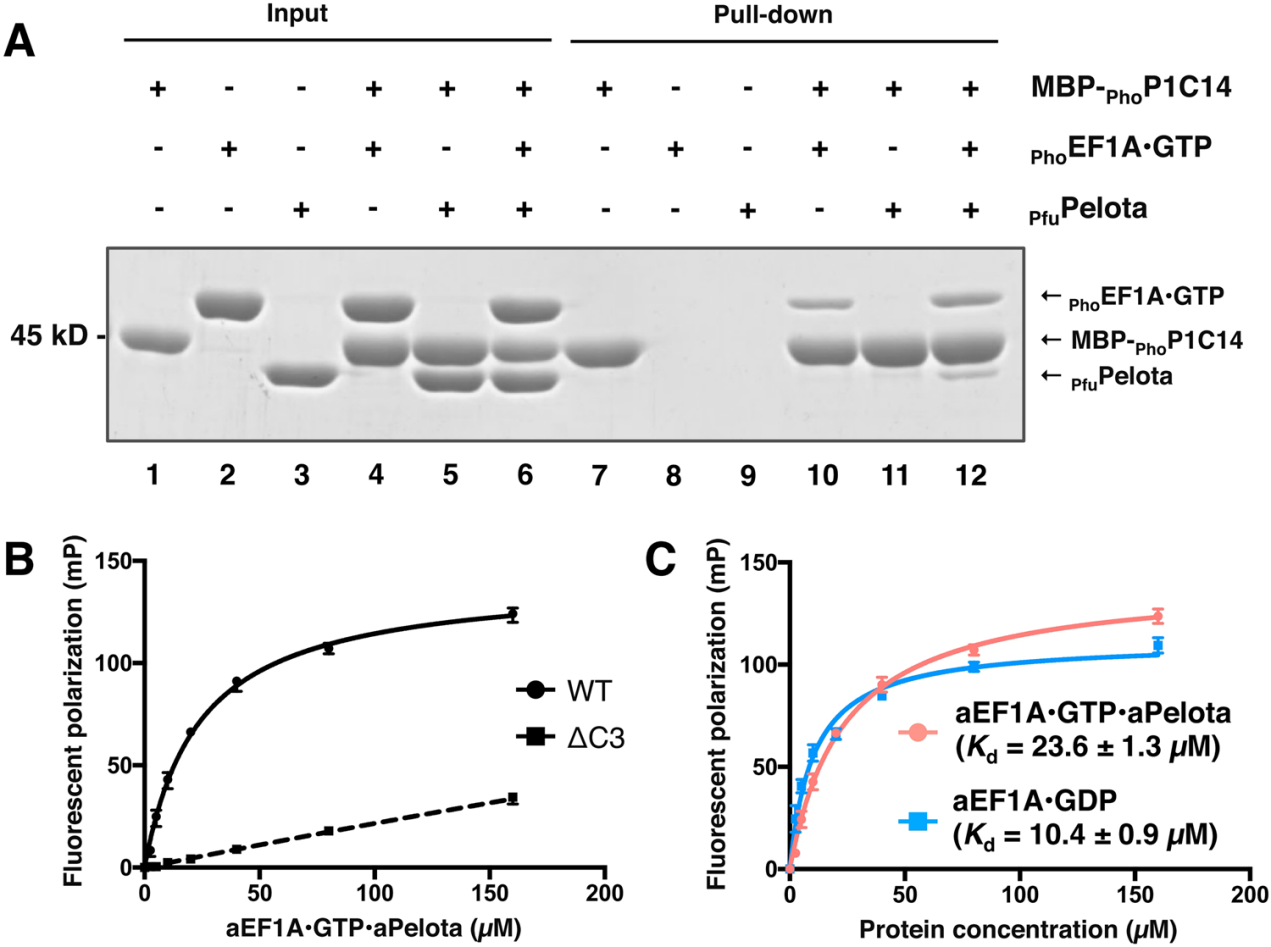
Biochemical analyses of the interaction between aEF1A•GTP and aP1. (**A**) *In vitro* pull-down assay using amylose resin. _Pho_EF1A, MBP-_Pho_P1C14, and _Pfu_Pelota indicated at the top of the gel were mixed without (lanes 1–6) or with amylose resin (lanes 7–12). A portion of input samples (lanes 1–6) and protein samples bound to amylose resin (lanes 7–12) were subjected to SDS-PAGE. The proteins were detected by CBB staining. For reference, the uncropped gel image is included as Fig. S9A. (**B**) Fluorescence polarization binding assay. The FITC-labeled peptide _Pho_P1C14 (WT) or _Pho_P1C14∆3 lacking the C-terminal 3 residues from _Pho_P1C14 (∆C3) was mixed with increasing concentrations of the _Pho_EF1A •GTP•_Pfu_Pelota complex, and mP values were determined for individual protein concentrations. Error bars represent SD values (n = 3). (**C**) The same Fluorescence polarization assay as (**B**), except that the binding of _Pho_P1C14 to the _Pho_EF1A•GTP•_Pfu_Pelota complex (red line) was compared with that of the _Pho_EF1A•GDP complex (blue line). The mP values were determined as in (**B**). The *K*_d_ values are presented under the curves.

Our previous study showed that _Pho_P1 binds to the _Pho_EF1A•GTP•aminoacyl-tRNA complex via its C-terminal region^12^. Therefore, we tested whether the C-terminal region of _Pho_P1 binds to the _Pho_EF1A•GTP•_Pfu_Pelota complex, using a pull-down assay with maltose-binding protein fused to the C-terminal 14 amino acid sequence of _Pho_P1 (MBP-_Pho_P1C14). As shown in Fig. 1A, MBP-_Pho_P1C14 co-precipitated not only with _Pho_EF1A•GTP (lane 10), but also with both _Pho_EF1A•GTP and _Pfu_Pelota (lane 12). However, _Pfu_Pelota was not precipitated in the sample lacking _Pho_EF1A•GTP (lane 11). The results indicate that the C-terminal region of _Pho_P1 binds to _Pho_EF1A within the _Pho_EF1A•GTP•_Pfu_Pelota complex. The binding between the C-terminal region and the _Pho_EF1A•GTP•_Pfu_Pelota complex was also detected by fluorescent polarization using a C-terminal 14-mer peptide of _Pho_P1, which was labeled with fluorescein isothiocyanate (FITC) at its N-terminus (Fig. 1B). Effective binding was not detected with a 11-mer peptide lacking the C-terminal 3 amino acids of the 14-mer peptide (_Pho_P1C14∆3). The *K*d value for the binding of the C-terminal peptide to the _Pho_EF1A•GTP•_Pfu_Pelota complex was estimated as 23.6 μM, a value which is comparable to the value of 10.4 μM determined for binding of the same C-terminal peptide to the _Pho_EF1A•GDP complex (Fig. 1C). The results indicate that the C-terminal region of _Pho_P1 binds both the _Pho_EF1A•GTP•_Pfu_Pelota and _Pho_EF1A•GDP complexes with a similar affinity.

### Structural basis for interaction of aP1 stalk protein with aEF1A•GTP•aPelota complex

**Figure 2:**
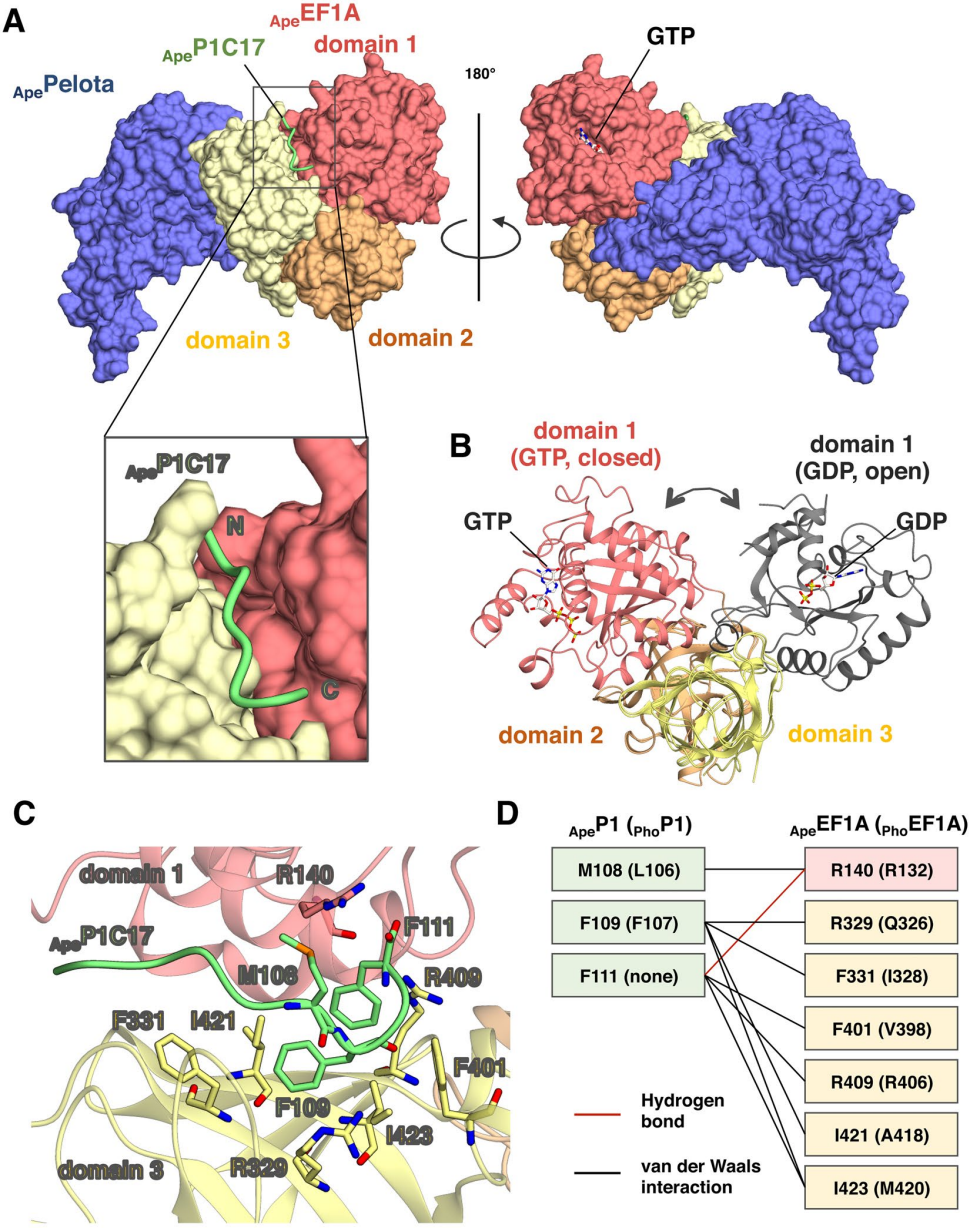
Crystal structure of the aP1-CTD•aEF1A•GTP•aPelota complex. (**A**) Overall structure of the GTP-bound complex _Ape_P1C17•_Ape_EF1A•GTP•_Ape_Pelota. _Ape_EF1A•_Ape_Pelota, _Ape_P1C17, and GTP are represented by surface, ribbon, and stick models, respectively. Colors in _Ape_EF1A are as follows, domain 1 (pink), domain 2 (orange) and domain 3 (yellow); _Ape_Pelota is blue; aP1 is green. The N- and C-termini of _Ape_P1C17 are indicated as ‘N’ and ‘C’, respectively. (**B**) Structural comparison of _Ape_EF1A•GTP in the complex described here with _Pho_EF1A•GDP [PDB ID: 3WY9]^12^ superposing domains 2 and 3. The colors of domains of aEF1A are as in (**A**), except for domain 1 of _Pho_EF1A•GDP (grey). (**C**) Structure of the binding interface between _Ape_P1C17 and _Ape_EF1A in the _Ape_P1C17•_Ape_EF1A•GTP•_Ape_Pelota complex. _Ape_EF1A and _Ape_P1C17 are represented by a ribbon model, and the amino acid residues of _Ape_EF1A and _Ape_P1C17 that participate in the interaction by stick models. The color coding is the same as in (**A**). (**D**) Schematic diagram of the interactions between _Ape_P1C17 and _Ape_EF1A. The van der Waals contacts and hydrogen bonds are represented by black and red lines, respectively. Amino acid residues and numbering are for *A*. *pernix* samples, and those in parentheses are for *P*. *horikoshii* samples.

To elucidate the molecular details of the interaction between the C-terminal region of aP1 and the GTP-bound form of aEF1A, we attempted crystallization of the P1•EF1A•GTP•Pelota tetrameric complex using samples from several species including *P*. *horikoshii*, *P*. *furiosus*, and *Aeropyrum pernix*. The results of these efforts were a crystal of the *A*. *pernix* tetrameric complex _Ape_P1C17•_Ape_EF1A•GTP•_Ape_Pelota that diffracted to 3.0 Å resolution. The data collection and structure refinement statistics are summarized in Tables S1 and S2. The quality of the electron density maps surrounding aP1 and GTP in the complex is shown in Fig. S2. The overall structure of the tetrameric complex is shown in Fig. 2A. As reported by Kobayashi *et al*.^16^, the orientation of domains 1, 2, and 3 of _Ape_EF1A•GTP in the presence of _Ape_Pelota is that of the closed state (Fig. 2B), as observed in the tRNA-bound form of bacterial EF-Tu•GTP^14^. A part of the _Ape_P1C17 peptide (S103 to F111) was observed in a hydrophobic space between domains 1 and 3 in an extended unstructured form (Fig. 2A). This P1 binding site was ~25 Å away from the GTP/GDP-binding site in domain 1.

Comparison of the structures of _Ape_P1C17-bound and _Ape_P1C17-free _Ape_EF1A•GTP•_Ape_Pelota revealed that, although the orientations of several amino acid side chains in the aP1 binding site of aEF1A differ markedly (details of the interaction between aEF1A and aP1 are described below), the overall structures of the two forms are highly similar (rms deviation of 0.49 Å for equivalent Cα atoms) (Fig. S3A,B). The conformations of regions including the P-loop and switch I and II elements, which are related to GTP hydrolysis, are also similar in the two structures (Fig. S3C). These results indicate that aP1 binding has no substantial effect on the overall structure of _Ape_EF1A including the GTP hydrolysis site in the domain 1.

### Detailed interaction between aP1 and the GTP-bound form of aEF1A

The structure of the _Ape_P1C17•_Ape_EF1A•GTP•_Ape_Pelota complex revealed that the three hydrophobic amino acids, M108, F109, and F111 of _Ape_P1 make contact with several amino acid residues of _Ape_EF1A in the complex (Fig. 2C,D). Thus, M108 binds to R140 in domain 1 of _Ape_EF1A via van der Waals interaction; F109 binds to R329, F331, I421, and I423 in domain 3 of _Ape_EF1A via van der Waals interactions; F111 binds to R140 via hydrogen bonding, and to F401, R409, and I423 in domain 3 of _Ape_EF1A via van der Waals interactions. It is particularly noteworthy that the F109 residue of _Ape_P1, which is highly conserved among eukaryotes/archaea (Fig. S4), is surrounded by hydrophobic amino acids (F331, I421, and I423 in the case of _Ape_EF1A) (Fig. 2C), as is the case with the binding to aEF1A•GDP, aEF2•GMPPCP, aIF5B•GDP, and aABCE1•ADP^12,19–21^. It is also notable that _Ape_P1 has an extra amino acid, F111, at its C-terminus, which is not present in other archaeal aP1 molecules (Fig. S4) and which does not seem to be essential for factor binding as described below.

### Functional effect of mutations at the aEF1A•stalk binding sites

**Figure 3:**
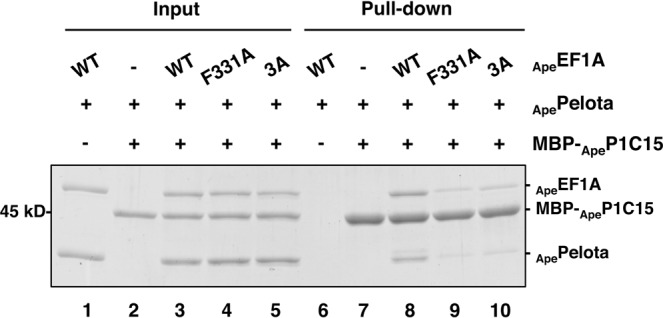
Effect of mutations at F331, I421, and I423 of _Ape_EF1A, which were identified as the binding site of the conserved F109 of _Ape_P1. *In vitro* pull-down assay using amylose resin. _Ape_EF1A, MBP-_Ape_P1C15, and _Ape_Pelota indicated at the top of the gel were mixed without (lanes 1–5) or with amylose resin (lanes 6–10). A portion of input samples (lanes 1–5) and protein samples bound to amylose resin (lanes 6–10) were subjected to SDS-PAGE. The proteins were detected by CBB staining. For reference, the uncropped gel image is included as Fig. S9B.

The crystal structural analysis indicates contact of M108, F109 and F111 of _Ape_P1 with _Ape_EF1A. The results are consistent with binding data shown in Fig. 1B, whereby deletion of the three C-terminal amino acids (L106, F107, and G108) of _Pho_P1 disrupted _Pho_EF1A binding. However, as described above, _Ape_P1 has an extra phenylalanine residue F111 at the C-terminus which is unique to _Ape_P1 (Fig. S4). We confirmed that deletion of F111 of _Ape_P1 resulted in no marked effect in binding to the _Ape_EF1A•GTP complex (Fig. S5). It is therefore likely that M108 and F109 of _Ape_P1 have the primary role in binding to _Ape_EF1A. Notably, the highly conserved F109 of _Ape_P1 binds to a hydrophobic pocket that is formed by the hydrophobic amino acids F331, I421, and I423 of _Ape_EF1A. To confirm the importance of this feature, we constructed a single mutant, F331A (the closest residue to F109 in _Ape_EF1A), and triple mutant F331A/I421A/I423 A (3A-mutant) of _Ape_EF1A, and tested their binding to _Ape_P1 (MBP-_Ape_P1C15) by pull-down assay (Fig. 3). Both the mutants associated with GTP and _Ape_Pelota showed a markedly reduced ability to bind to MBP-_Ape_P1C15 (lanes 9 and 10), compared with the wild type _Ape_EF1A (lane 8).

**Figure 4:**
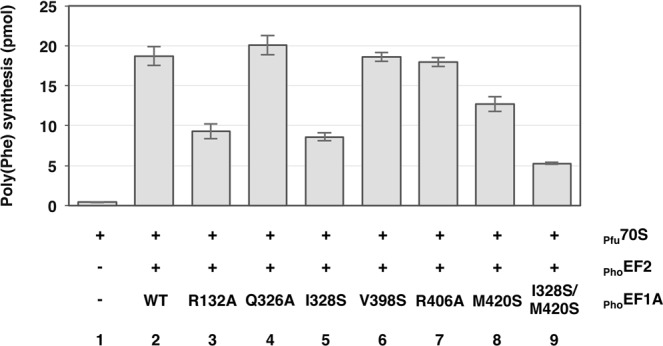
Effects of the amino acid substitutions in aEF1A on aEF1A/aEF2-dependent polyphenylalanine synthesis. *P*. *furiosus* 70S ribosomes were incubated with *P*. *horikoshii* aEF2 and *P*. *horikoshii* wild-type aEF1A (bar 2) or each of the aEF1A mutants (bars 3–9) in the presence of poly(U) and *E*. *coli* [^14^C]Phe-tRNA. The amount of polyphenylalanine synthesized in the 10 min reaction was assayed as described in Materials and Methods. Error bars represent SD values (n = 3).

The present structural study showed that 7 amino acid residues (R140, R329, F331, F401, R409, I421, and I423) of _Ape_EF1A interact with the C-terminal region of _Ape_P1 (Fig. 2). Amino acid residues at these positions are conserved in archaea at least in terms of their properties (Fig. S6). Indeed, it is remarkable that R409 is highly conserved from archaea to eukaryotes. We next investigated the effect of mutations of these amino acid residues of EF1A on translation elongation. By using *P*. *horikoshii* elongation factors aEF1A/aEF2 with *P*. *furiosus* 70 S ribosomes, we have previously established a high temperature polyphenylalanine synthesis system^13^. Here we introduced six mutations in *P*. *horikoshii* EF1A, namely at R132, Q326, I328, V398, R406, and M420, which correspond to R140, R329, F331, F401, R409, and I423 in _Ape_EF1A, respectively. In the present crystal structure, all these amino acid residues interacts with _Ape_P1 via their side chains (A418 of _Pho_EF1A, which corresponds to I421 of _Ape_EF1A, was excluded from the mutagenesis study because it is an alanine residue). We then tested these mutants for poly(U)-dependent polyphenylalanine synthesis activity (Fig. 4). The R132A and I328S point mutations and I328S/M420S double mutation (which are presumed to interact with F107 of _Pho_P1, a highly conserved phenylalanine residue) caused considerable reduction in activity, while the M420S mutation resulted in only slightly reduced activity. In addition, the effects of these mutations of _Pho_EF1A were consistent with data on binding between _Pho_EF1A•GTP•_Pfu_Pelota and _Pho_P1C14 peptide, assayed by fluorescent polarization (Table S3). Although the effects of the point mutations on the binding assay were small, the results were reproducible. These biochemical analyses support the crystal structural data presented here.

## Discussion

The ribosomal stalk protein plays an important role in the recruitment of the GTP-bound form of elongation factor EF-Tu in bacteria, eEF1A in eukaryotes, and aEF1A in archaea, and thus in the delivery of the associated aminoacyl-tRNA to the A site of the ribosome. It had been expected that the stalk binding mode for GTP-bound aEF1A would differ from that for the GDP-bound form which has been reported previously^12^, because the relative location of domain 1 of aEF1A would be expected to differ in the closed GTP-bound and open GDP-bound conformations. In the present study using archaeal samples, we show that aP1-CTD binds to aEF1A•GTP with similar affinity to aEF1A•GDP. Furthermore, we have determined the crystal structure of the complex of the C-terminal aP1 peptide with aEF1A•GTP•aPelota, which has revealed that the binding mode of aP1 changes markedly upon the conformational switch between the closed and open conformations of aEF1A.

In the aP1-CTD•aEF1A•GTP•aPelota complex, an extended form of the aP1 peptide bound to the interface of domains 1 and 3 of aEF1A in the GTP-bound closed conformation (Fig. 2). This observation is supported by binding experiments (Fig. 3 and Table S3). All mutations of the putative aP1-binding site of aEF1A, which reduced the binding of aP1 to aEF1A•GTP, also decreased the ribosome-dependent polyphenylalanine synthesis (Fig. 4). Therefore, it is likely that the binding of aP1 to aEF1A•GTP as described in this study is functionally important and presumably contributes to recruitment of the ternary complex of aEF1A•GTP•aminoacyl-tRNA to the ribosome. It is interesting and worthwhile to consider the significance of why aP1 binds to the interface of domains 1 and 3 in aEF1A•GTP. A recent study using fluorescence resonance energy transfer (FRET) showed that EF-Tu•GTP is not locked in the closed conformation in solution, but adopts a dynamic conformation in which domain 1 can swing, even in the ternary complex with aminoacyl-tRNA^18^. Furthermore, this study also showed that the ribosomal factor binding center selectively accepts the EF-Tu•GTP•aminoacyl-tRNA complex in the closed conformation^18^. Based on these findings, we infer that aP1 facilitates the formation of the closed conformation of aEF1A•GTP by bridging domains 1 and 3, probably with the aid of aminoacyl-tRNA/aPelota/aRF1, and promotes the selective binding of the closed-conformation aEF1A•GTP•aminoacyl-tRNA/aPelota/aRF1 complex to the factor binding center of the ribosome.

**Figure 5:**
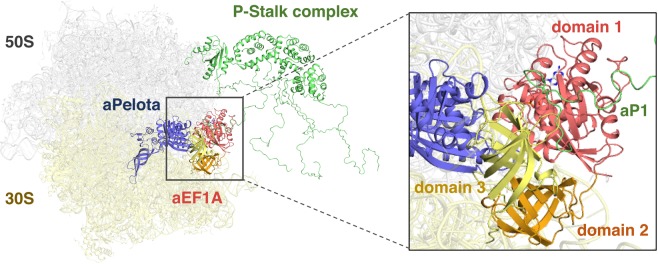
Model of aP1•aEF1A•GTP•aPelota complex docking with the 70S ribosome. Colors are as follows: 50S (white), 30S (light yellow), stalk complex (green), aPelota (blue), aEF1A domain 1 (pink), aEF1A domain 2 (orange) and aEF1A domain 3 (yellow). In this docking model, we superimposed the aP1•aEF1A•GTP•aPelota complex described in this paper onto the bacterial 70 S•EF-Tu•GDPCP•aminoacyl-tRNA complex [PDB ID: 4V5G]^22^ (Voorhees *et al*. 2010). Subsequently the aP0•aP1 stalk complex core from *Pyrococcus horikoshii* [PDB ID: 3A1Y]^10^ was superimposed onto the model. The aP0•aP1 stalk complex core and aP1C17 peptide bound to aEF1A were connected by the flexible hinge region of aP1. Other hinge regions of aP0 and aP1 were modeled arbitrarily.

To reinforce the idea that the binding of the C-terminal region of aP1 facilitates factor recruitment to the factor binding center of the ribosome, we constructed a docking model, using the structures of the bacterial 70 S ribosome•EF-Tu•GDPCP•aminoacyl-tRNA complex^22^ and the archaeal heptameric stalk protein complex^10^ (Fig. 5). The model shows that the aP1-binding site within aEF1A is facing outward, and that the C-terminal region of aP1 can reach the ribosome-associated aEF1A•GTP via the flexible C-terminal half of aP1 without steric hindrance. Thus, this docking supports our view that aP1 directly binds to aEF1A via the C-terminal region of aP1, and contributes to the recruitment of aEF1A to the factor binding center of the ribosome.

**Figure 6:**
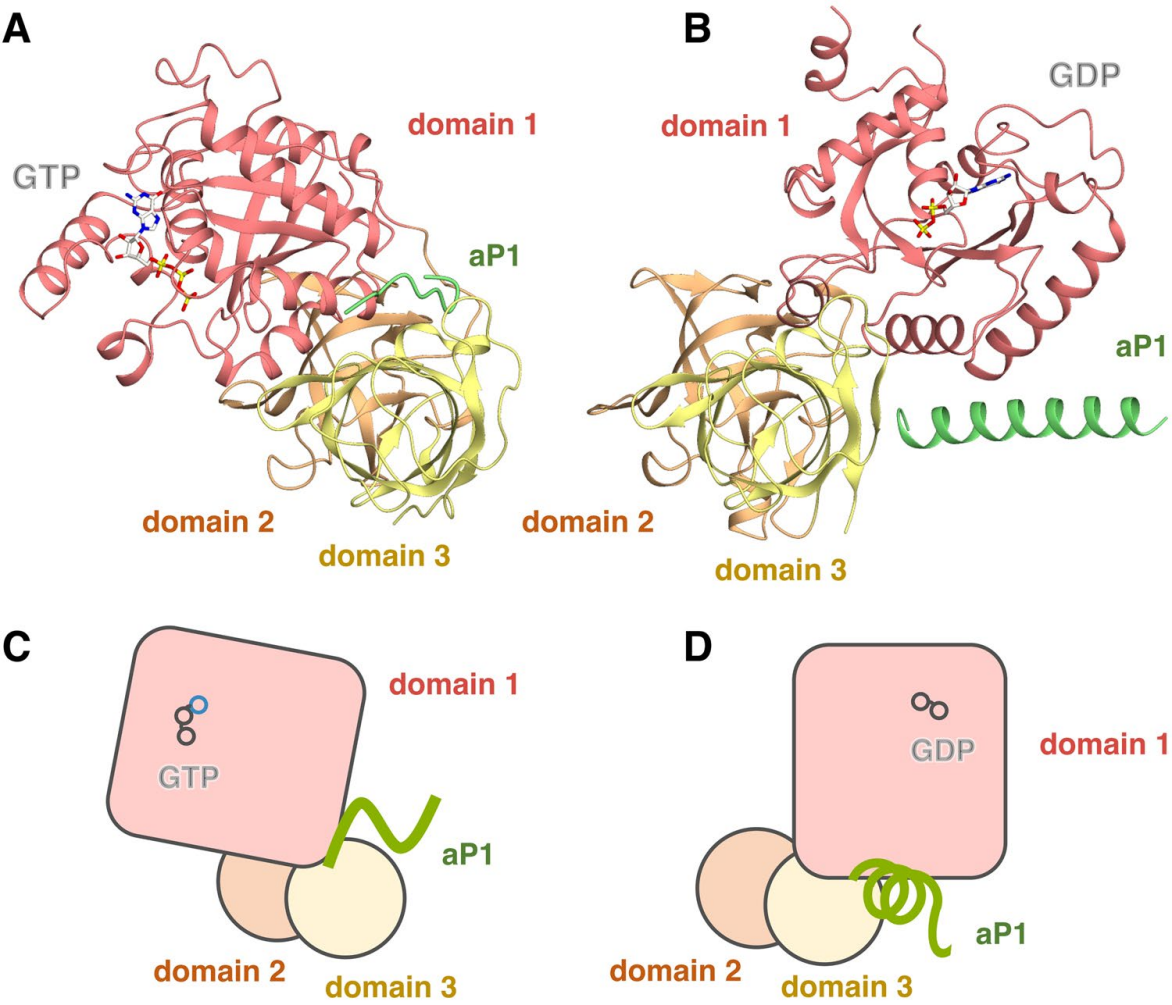
Comparison of the structures of the GTP- and GDP-bound forms of aP1•aEF1A complexes. Comparison of crystal structures of aP1•aEF1A•GTP (**A**,**C**) and aP1•aEF1A•GDP (**B**,**D**) [PDB ID: 3WY9]^12^. Schematic representations of the interaction in the GTP-bound and GDP-bound forms of aP1•aEF1A complexes are shown in (**C**,**D**), respectively. Panel (A,B) are shown from the same orientation and direction with respect to domains 2 and 3.

Comparison of the current results describing aP1 binding to the GTP-bound form of aEF1A with previous results that describe binding to the GDP-bound form demonstrates that as expected the binding modes differ greatly in the two cases (Figs 6 and S7). In the results described here, the extended C-terminal peptide of aP1 binds to the interface of domains 1 and 3 of the closed GTP-bound conformation of aEF1A and this involves 3 residues of aP1 and 7 residues of aEF1A (Fig. S7). In the aP1•aEF1A•GDP, the C-terminal 27 residues of aP1 form an α-helix, and binds to a different site of the interface between domains 1 and 3 in the opened GDP-bound form of aEF1A, via 8 residues of aP1 and 9 residues of aEF1A (Fig. S7). Of these residues, only the conserved M108 and F109 of _Ape_P1 (L106 and F107 in _Pho_P1, respectively) are involved in binding of aP1 to both aEF1A•GDP and to aEF1A•GTP (Fig. S7). These findings suggest that the flexible C-terminal region of aP1 can bind to the two conformations of aEF1A in the process of GTP hydrolysis (aEF1A•GTP and aEF1A•GDP), and that it adopts an induced conformation dependent on the structures of aEF1A (Figs 6 and S8). This presumption is applicable to stalk binding to the other translational GTPases aIF5B^20^, aEF2^19^, and the translational ATPase aABCE1^21^. These crystal structural analyses have revealed that the conformation of the C-terminal region of aP1 in these complexes differs (Fig. S8). The variable structural properties of the C-terminal region which depends on associated proteins seems to be related to the functional role of the stalk proteins in efficient recruitment of various translational GTP/ATPases to the ribosome in translation.

The question arises as to why aP1 maintains the binding to switched GTP/GDP-bound conformations of aEF1A, and we propose a hypothesis as follows. As described above, the binding of aP1 to the GTP-bound form of aEF1A would promote the selective binding of the closed-conformation aEF1A•GTP•aminoacyl-tRNA/aPelota/aRF1 complex to the factor binding center of the ribosome. Upon the binding, GTP hydrolysis occurs and the conformational change of aEF1A from the closed conformation to the open conformation takes place on the ribosome. In this process, aP1 would temporarily release aEF1A, and subsequently aP1 could bind again to aEF1A to stabilize, in turn, the open GDP-bound conformation by bridging domains 1 and 3 in a different position from that in aEF1A•GTP (Figs 6 and S7). Because aEF1A•GDP is released form the ribosome in the open conformation, this stabilization of the open conformation of aEF1A by aP1 would promote the release of aEF1A•GDP from the ribosome. Furthermore, we infer that the switching of the binding of the stalk mentioned above also takes place in eukaryotes (eEF1A•GTP/GDP) and eubacteria (EF-Tu•GTP/GDP). Further studies are needed to validate these hypotheses.

## Methods

### Plasmid construction

The coding sequences for elongation factor aEF1A of the hyperthermophilic archaeon *Aeropyrum pernix* (_Ape_EF1A) were amplified from the genome by PCR, and inserted between the *Nde*I and *Xho*I sites in the *E*. *coli* expression vector, pET-15b containing a six His-tag. The coding sequences for aPelota were amplified from *Pyrococcus furiosus* and *A*. *pernix* genomes, and inserted between the *Nde*I and *Bam*HI sites in the pET-15b vector. The plasmid for expression of *P*. *horikoshii* aEF1A (_Pho_EF1A) and aEF2 (_Pho_EF2) were as described previously^12,23^. The plasmid for expression of N-terminal maltose binding protein (MBP) fused with the C-terminal 14 amino acid residues of *P*. *horikoshii* aP1 (MBP-_Pho_P1C14) was constructed by removing the coding sequence for residues 61–94 from pMAL-c4x-aP1[61–108]^21^ by PCR. Although pMAL-c4x-aP1[61–108] was prepared using the *P*. *furiosus* genome, the amino acid sequence for the C-terminal 14 amino acid residues of *P*. *furiosus* aP1 is identical to that of *P*. *horikoshii* aP1 (Fig. S4). The plasmid for expression of N-terminal MBP fused with the C-terminal 15 amino acid residues of *A*. *pernix* aP1 (MBP-_Ape_P1C15) was constructed by insertion of the PCR-amplified sequence corresponding to the C-terminal 15 amino acid residues of *A*. *pernix* aP1 between the *Eco*RI and *Pst*I sites in the pMAL-c4x vector. Site-directed mutagenesis of _Pho_EF1A or _Ape_EF1A was performed by PCR using the plasmid carrying each EF1A gene, as described previously^12^. The PCR primers are listed in Table S4.

### Protein expression and purification

_Ape_EF1A, _Pho_EF1A, their mutants, _Pfu_Pelota, _Ape_Pelota, and the fusion proteins MBP-_Pho_P1C14 and MBP-_Ape_P1C15 were all expressed in *E*. *coli* BL21 (DE3) codon plus RIL or BL21 (DE3) by adding 0.5 mM IPTG. After incubation at 37 °C for 3 h (for _Pho_EF1A, its mutants, and MBP-_Pho_P1C14) or 20 °C for 18 h (for _Ape_EF1A, its mutants, _Ape_Pelota, _Pfu_Pelota, and MBP-_Ape_P1C15), cells were harvested by centrifugation, resuspended in Lysis buffer (20 mM HEPES-KOH pH7.6, 1 M NH_4_Cl, 5% (v/v) glycerol, 7 mM 2-ME) and disrupted by sonication. After sedimenting cell debris and other insoluble materials, _Pho_EF1A, _Ape_EF1A and their mutants were purified by heat treatment (80 °C for 20 min) of cell extracts, Ni-affinity chromatography and size-exclusion chromatography. The His-tag of aEF1A samples and its mutants was cleaved with thrombin before anion-exchange chromatography. To prepare the GTP-bound form of aEF1A, tightly bound GDP was removed from purified aEF1A by bacterial alkaline phosphatase treatment, as described previously^12^. MBP-_Pfu_P1C14 and MBP-_Ape_P1C15 were purified by amylose-affinity chromatography and size-extraction chromatography. _Ape_Pelota and _Pfu_Pelota were purified by heat treatment (70 °C for 20 min) of cell extracts, Ni-affinity chromatography and anion-exchange chromatography.

All peptides used were synthesized by Hokkaido System Science Co., Ltd (Sapporo, Japan). The C-terminal 17-mer peptide of *A*. *pernix* aP1 (_Ape_P1C17) [sequence: KKEEEVDLSGLSGMFGF] was used for crystallization (See Fig. S4). The C-terminal 14-mer peptide of *P*. *horikoshii* aP1 (_Pho_P1C14) [sequence: SEEEALAGLSALFG] and the 11mer, the same peptide as the 14-mer, but lacking the C-terminal 3 residues (_Pho_P1C14∆3) [sequence: SEEEALAGLSA], were labeled with fluorescein isothiocyanate (FITC) at the N-termini and used for binding experiments.

### Crystallization and structure determination

Crystals of the _Ape_P1C17•_Ape_EF1A•GTP•_Ape_Pelota complex were prepared by the sitting drop vapor diffusion method at 20 °C by mixing 1 µl of protein solution (1.55 mg/ml _Ape_P1C17 peptide, 6.5 mg/ml _Ape_EF1A, 5.4 mg/ml _Ape_Pelota, 2.5 mM GTP, 25 mM Tris-HCl, pH 8.0, 500 mM NaCl, 5 mM MgCl_2_, 1 mM DTT) and an equal volume of reservoir solution (100 mM Tris-HCl, pH 8.0, 200 mM Li_2_SO_4_, 16% (w/v) PEG3350). Diffraction data were collected with a wavelength of 1.000 Å at 95 K on the BL-5A of the Photon Factory (Tsukuba, Japan) using an ADSC Quantum 315r CCD detector. Diffraction data were processed with the program HKL2000 (HKL Research). The initial model of the _Ape_P1C17•_Ape_EF1A•GTP•_Ape_Pelota complex was obtained by the molecular replacement method using MOLREP^24^, using the structures of _Ape_EF1A•GTP•_Ape_Pelota [PDB ID: 3WXM]^16^ as a search model. The model was then manually refined using COOT and REFMAC5^25,26^. The resultant data collection and refinement statistics are summarized in Supplementary Tables S1 and S2. Structural figures were prepared using CueMol2 (http://www.cuemol.org/en/) and PyMOL (https://pymol.org/2/).

### Pull-down assay

EF1A (1,000 pmol) and Pelota (1,000 pmol) were mixed with equal amounts of MBP-P1 in 100 µl of PD buffer (25 mM Tris-HCl pH 8.0, 200 mM NaCl, 10 mM MgCl_2,_ 1 mM GTP). Subsequently, 20 µl of amylose resin (New England Biolabs) was added to the mixture. After incubation with rotation at 4 °C for 90 min, the beads were washed with PD buffer and then bound proteins were co-eluted with 50 µl of PD buffer containing 50 mM maltose. The eluted proteins were analyzed by SDS-PAGE, followed by CBB staining^21^.

### Fluorescent polarization binding assay

The FITC-labeled peptide (50 nM) was mixed with 0, 2.5, 5, 10, 20, 40, 80, 160 µM of the _Pho_EF1A•GTP•_Pfu_Pelota, or _Ape_EF1A•GTP•_Ape_Pelota complex in 100 µl of FP buffer (20 mM HEPES-KOH pH 7.6, 10 mM MgCl_2_, 150 mM KCl, 0.1 mg/ml BSA). All measurements were performed at 25 °C using a Pan Vera Beacon 2000 fluorescence polarization instrument (Invitrogen), as described in the manufacturer’s protocol. Curve fitting was performed using nonlinear regression using saturation binding–one site specific binding in GraphPad Prism 6, GraphPad Software (San Diego, California USA).

### Polyphenylalanine synthesis

*P*. *furiosus* ribosomes were prepared, as described previously^27^, except the buffer for preparation and storage contained 0.5 mM spermine. Poly(U)-dependent polyphenylalanine synthesis was assayed using the isolated ribosomes, _Pho_EF1A/_Pho_EF2, and [^14^C]Phe-tRNA, as described previously^13^. Functional effects of mutations of _Pho_EF1A were tested on the polymerization activity.

## Supplementary information

Supplementary Information

## Data Availability

The structural coordinates of the refined model and the structure factors have been deposited in the RCSB Protein Data Bank (www.rcsb.org/) and assigned the identifier 6JI2. All other data supporting the findings of this study are available within the paper and Supplementary Information files.

## References

[1] Rodnina MV, Wintermeyer W (2009). Recent mechanistic insights into eukaryotic ribosomes. Curr. Opin. Cell Biol..

[2] Schmeing TM, Ramakrishnan V (2009). What recent ribosome structures have revealed about the mechanism of translation. Nature.

[3] Liljas, A. & Ehrenberg, M. The catalysts––translation factors. *Structural Aspects of Protein Synthesis* (2^nd^ edition). 149–228 (World Scientific, Singapore, 2013).

[4] Stark H (2002). Ribosome interactions of aminoacyl-tRNA and elongation factor Tu in the codon-recognition complex. Nat. Struct. Biol..

[5] Saito K (2010). Omnipotent role of archaeal elongation factor 1 alpha (EF1α) in translational elongation and termination, and quality control of protein synthesis. Proc Natl Acad Sci USA.

[6] Liljas A, Sanyal S (2018). The enigmatic ribosomal stalk. Q. Rev. Biophys..

[7] Mohr D, Wintermeyer W, Rodnina MV (2002). GTPase activation of elongation factors Tu and G on the ribosome. Biochemistry.

[8] Diaconu M (2005). Structural basis for the function of the ribosomal L7/12 stalk in factor binding and GTPase activation. Cell.

[9] Maki Y (2007). Three binding sites for stalk protein dimers are generally present in ribosomes from archaeal organism. J. Biol. Chem..

[10] Naganuma T (2010). Structural basis for translation factor recruitment to the eukaryotic/archaeal ribosomes. J. Biol. Chem..

[11] Nomura N (2012). Archaeal ribosomal stalk protein interacts with translation factors in a nucleotide-independent manner via its conserved C terminus. Proc. Natl. Acad. Sci. USA.

[12] Ito K (2014). Molecular insights into the interaction of the ribosomal stalk protein with elongation factor 1α. Nucleic Acids Res..

[13] Imai H (2015). Functional role of the C-terminal tail of the archaeal ribosomal stalk in recruitment of two elongation factors to the sarcin/ricin loop of 23S rRNA. Genes Cells.

[14] Nissen P (1995). Crystal structure of the ternary complex of Phe-tRNA^Phe^, EF-Tu, and a GTP analog. Science.

[15] Polekhina G (1996). Helix unwinding in the effector region of elongation factor EF-Tu–GDP. Structure.

[16] Kobayashi K (2010). Strucural basis for mRNA surveillance by archaeal Pelota and GTP-bound EF1α complex. Proc. Natl. Acad. Sci. USA.

[17] Kobayashi K, Saito K, Ishitani R, Ito K, Nureki O (2012). Structural basis for translation termination by archaeal RF1 and GTP-bound EF1α complex. Nucleic Acids Res..

[18] Johansen JS (2018). *E*. *coli* elongation factor Tu bound to a GTP analogue displays an open conformation equivalent to the GDP-bound form. Nucleic Acids Res..

[19] Tanzawa T (2018). The C-terminal helix of ribosomal P stalk recognizes a hydrophobic groove of elongation factor 2 in a novel fashion. Nucleic Acids Res..

[20] Murakami R (2018). The interaction between the ribosomal stalk proteins and translation initiation factor 5B promotes translation initiation. Mol. Cell Biol..

[21] Imai H (2018). The ribosomal stalk protein is crucial for the action of the conserved ATPase ABCE1. Nucleic Acids Res..

[22] Voorhees RM, Schmeing TM, Kelley AC, Ramakrishnan V (2010). The mechanism for activation of GTP hydrolysis on the ribosome. Science.

[23] Nomura T (2006). *In vitro* reconstitution of the GTPase-associated centre of the archaebacterial ribosome: the functional features observed in a hybrid form with Escherichia coli 50S subunits. Biochem. J..

[24] Vagin A, Teplyakov A (1997). MOLREP: an automated program for molecular replacement. J. Appl. Crystallogr..

[25] Emsley P, Cowtan K (2004). Coot: model-building tools for molecular graphics. Acta Crystallogr. D Biol. Crystallogr..

[26] Murshudov GN (2011). REFMAC5 for the refinement of macromolecular crystal structures. Acta Crystallogr. D Biol. Crystallogr..

[27] Benelli D, Londei P (2007). *In vitro* studies of archaeal translational initiation. Methods Enzymol..

