# Supplementary material for: Switch of the interactions between the ribosomal stalk and EF1A in the GTP- and GDP-bound conformations
Source: Sci Rep. 2019 Oct 14;9:14761. doi: 10.1038/s41598-019-51266-x (PMC6791870; doi:10.1038/s41598-019-51266-x)
Supplement: Supplementary file 1 — Supplementary Information [file 41598_2019_51266_MOESM1_ESM.pdf]

Supplementary Information

Switch of the interactions between the ribosomal stalk and EF1A in the GTP- and GDP-bound conformations

Kei Maruyama[1,3], Hiarotatsu Imai[1,3], Momoko Kawamura[1], Sonoko Ishino[2], Yoshizumi Ishino[2], Kosuke Ito[1,4], and Toshio Uchiumi[1,4]

[1]Department of Biology, Faculty of Science, Niigata University, Ikarashi 2-8050, Nishi-ku, Niigata 950-2181, Japan
[2]Department of Bioscience and Biotechnology, Graduate School of Bioresource and Bioenvironmental Sciences, Kyushu University, Fukuoka 812-8581, Japan

[3]Contributed equally
[4]Correspondence and requests for materials should be addressed to
T.U. (uchiumi@bio.sc.niigata-u.ac.jp)
K.I. (k-ito@bio.sc.niigata-u.ac.jp)

Supplementary Figures

```
              ************:***.*************: ************:***********.:*****:*.****:*:******
P_furiosus    MEILEEKPKEGKIKIKAETLDDLWHLYHIISEGDVVYAKTLRKQAQRSDSLRPEKVEAVPVFLGIKAEKINLHRFANQLR    80
P_horikoshii  MEILEEKPKEGKVKIKVETLDDLWHLYHIITPGDVVYAKTLRKQSQRSDSLRPEKVEVIPVFLGVKVEKINFHKFANQLR    80

              :***************:***::*****:**:***:** *:*******:*:**:*:****:*******:**********:.
P_furiosus    ITGPIIYASREDVPLGRYHTLTVEPGTVITIQKEKWKNYHIERLKEAIESSKKARVMIVAIEDGEAEIAIVREYGLDFVG   160
P_horikoshii  VTGPIIYASREDVPLGKYHTIAVEPGTIITLQKERWKPYYIERLKEAVEASKRAKVMIVTIEDGEAEMAIVREYGLDFIA   160

              :* :*:.********:***:****:***:*:::*.****::***********:* :**:****:**.*:* ********.
P_furiosus    SITYNISGKRYNIKRDDEEKKFFHEVAKSMEELMKRENIEKAIVAGPGFYKENFVNFLRENYPELAKKVVTDDTSMGGRT   240
P_horikoshii  TIRHNLGGKRYNIKREDEERKFFHDVAKTMKDVMSRENIQRAIVAGPGFYKEDFYKFLKENYPDLASKIVLDDTSMGGRV   240

              ***************:****::**********.***:  ****:******.****** *:***.****: ***:******
P_furiosus    GIYEVIKRGTVDKVYTESRISKEIKLVEKVIEEIAKNGLVAYGLKEVEEATNYGAVETLIVLDSLLKGELREKIEELMEL   320
P_horikoshii  GIYEVIKRGTVDKVYSESRIANEIKLVEKVIERIAKDEPVAYGMKEVEEAVNYGAVEILLVLDELLKGDNREKVEELMEL   320

              **.**:.***********:*********.:******
P_furiosus    ARNLRASVVVVSSEHEGGDKLKALGGIAALLRFKIK   356
P_horikoshii  ARSLRSKVVVVSSEHEGGEKLKALGGIAGILRFKIK   356
```

Figure S1. Amino acid sequence alignment of Pelota from *Pyrococcus furiosus* (P_furiosus) and *Pyrococcus horikoshii* (P_horikoshii). The sequences were aligned using the program Clustal W (http://www.genome.jp/tools-bin/clustalw). Clustal X (http://www.clustal.org/clustal2/) was used to prepare the figure.

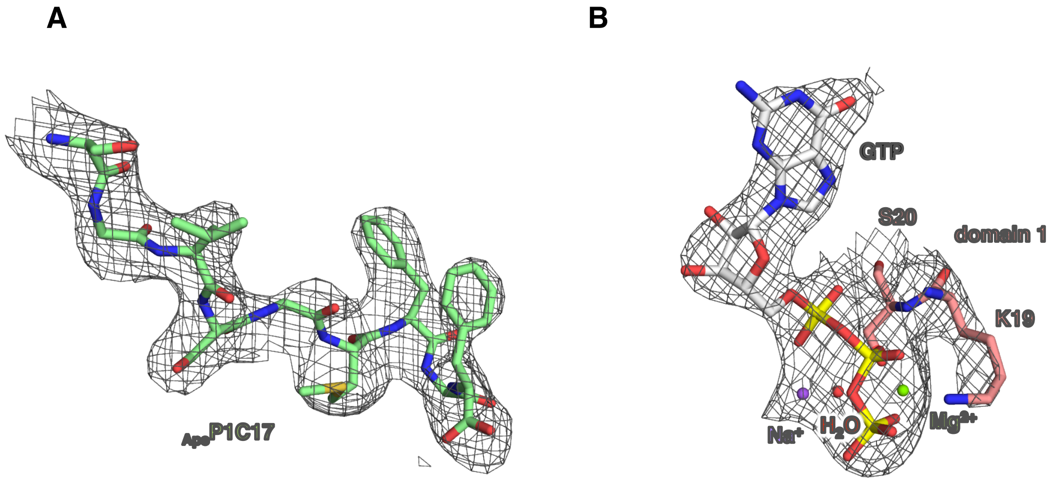

Figure S2. Quality of the electron density maps surrounding aP1 (A) and GTP (B) in the $_{Ape}$P1C17•$_{Ape}$EF1A•GTP•$_{Ape}$Pelota complex structure. The $2Fo - Fc$ electron density maps are shown. The color-coding is the same as in Fig. 2. In panel B, the parts of the electron density surrounding the phosphate moieties of GTP are fused with those of neighboring residues, Na^+/Mg^{2+} ions, and water molecule, probably due to the low resolution of the diffraction data. Therefore, to construct the model around the phosphate moieties, we used the high resolution crystal structures of $_{Ape}$EF1A [PDB ID: 3WXM (Kobayashi et al., 2010)] and $_{Cte}$IF5B [PDB ID: 4TMW (Kuhle et al., 2014)] as reference models.

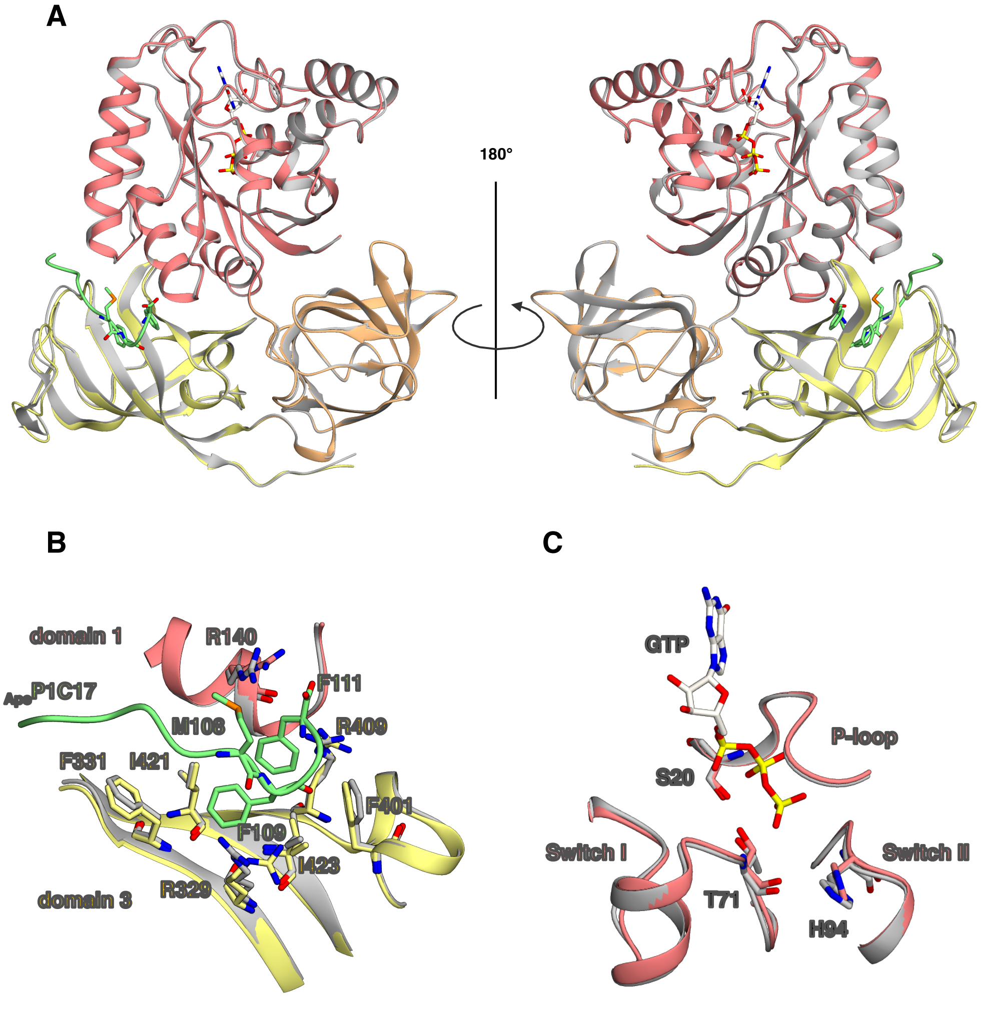

Figure S3. Effect of aP1 binding on the structure of $_{Ape}$EF1A•GTP•$_{Ape}$Pelota. Only the structure of $_{Ape}$EF1A•GTP is shown here. The structure of $_{Ape}$P1C17•$_{Ape}$EF1A•GTP complex (The color-coding is the same as in Fig. 2) was superimposed onto that of the $_{Ape}$EF1A•GTP complex (grey) [PDB ID: 3WXM] (Kobayashi et al., 2010). A, the overall structures; B, aP1-binding site; C, the key regions for GTP hydrolysis. In panel B, residues involved in the binding of aP1 are represented by a stick model. In panel C, residues involved in GTP Hydrolysis are represented by a stick model.

```
                           : :* :      :  : :     *  :  :      .. :*   *: .::    :        ...
P_furiosus     --------MEYVYAALLLHSVGKEINEENLKAVLQAAGVEPDEARIKALVAALEGVNIDEVIE-KAAMP----VAVAAAP   67
P_horikoshii   --------MEYVYAALLLHSVGKEINEENLKAVLQAAGVEPEEARIKALVAALEGVNIDEVIE-KAAMP----VAVAAAP   67
S_solfataricus --------MEYIYASLLLHSAKKEISEDALKNVLTAAGISVDEVRLKAVVAALKEVNIDEVLKNAAAMP----VAVAAQP   68
A_pernix       ---MAQEGKAYIHLALAIYYAGGKIDEETLKKAAEAIGMQVDEAKIKMLVASLEEVNLEEVLKQAVAAP-------VAAAA   71
H_sapines      --MASVSELACIYSALILHDDEVTVTEDKINALIKAAGVNVEPFWPGLFAKALANVNIGSLICNVGAG------GPAPAAG   73
B_mori         --MVSKAELACVYSALILVDDDVAVTGEKISTILKAAAVDVEPYWPGLFAKALEGINVRDLITNIGSG----VGAAPAAG   74
S_cerevisiae   --MSTESALS--YAALILADSEIEISSEKLLTLTNAANVPVENIWADIFAKALDGQNLKDLLVNFSAG-------AAAPAG   70

                *               * : * :::       **       .:*.
P_furiosus     AAAP---AGGG-EEKKEEEKKEEEEKEEE--VSEEEALAGLSALFG-   107
P_horikoshii   AAAP---AEAGGEEKKEEEKKEEEEKEEE--VSEEEALAGLSALFG-   108
S_solfataricus QATQ---AQPAAEEKKEE-KKEEEKKGP----SEEEIASGLASLFG-   106
A_pernix       AAPA---AAPAAEEKAEEEKKEEEEEKKE----EEVDLSGLSGMFGF   111
H_sapines      AAPAGGPAPSTAAAPAEEKKVEAKKEE-----SEESDDDMGFGLFD-   114
B_mori         GAPA---AAAAAGAPAEEKKEEKKEEE-----PEESDDDMGFGLFD-   112
S_cerevisiae   VAGG------VAGGEAGEAEAEKEEEEA----KEESDDDMGFGLFD-   106
```

Figure S4. Amino acids sequence alignment of aP1. The sequences of amino acids for Archaeal aP1 from *Pyrococcus furiosus* (P_furiosus), *Pyrococcus horikoshii* (P_horikoshii), *Sulfolobus solfataricus* (S_solfataricus), and *Aeropyrum pernix* (A_pernix), and for eukaryotic P1 from *Homo sapiens* (H_sapiens), *Bombyx mori* (B_mori), and *Saccharomyces cerevisiae* (S_cerevisiae) were aligned using the program Clustal W (http://www.genome.jp/tools-bin/clustalw). Clustal X (http://www.clustal.org/clustal2/) was used to prepare the figure.

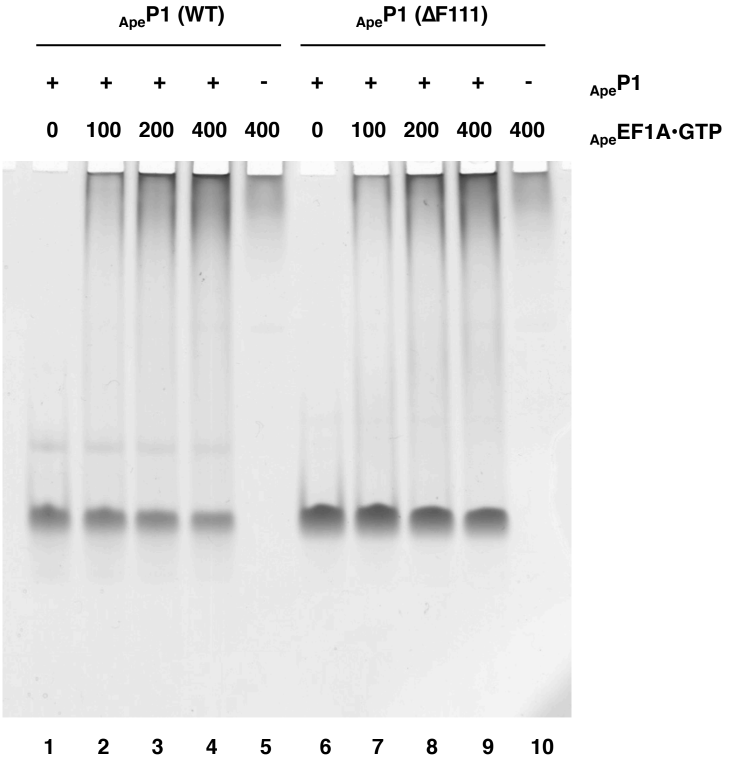

Figure S5. No effect of deletion of C-terminal F111 of $_{Ape}$P1 on binding to the $_{Ape}$EF1A•GTP complex. $_{Ape}$P1 (200 pmol) (WT) or the same amount of $_{Ape}$P1 lacking F111 (ΔF111) were incubated with 100, 200, and 400 pmol of $_{Ape}$EF1A•GTP complex (lanes 2–4 and 7–9) or without the complex (lanes 1 and 6). Lanes 5 and 10, 400 pmol of the complex alone. All samples were subjected to Native-PAGE as described previously (Nomura et al., 2012). For reference, the uncropped gel image is included as Figure S9C.

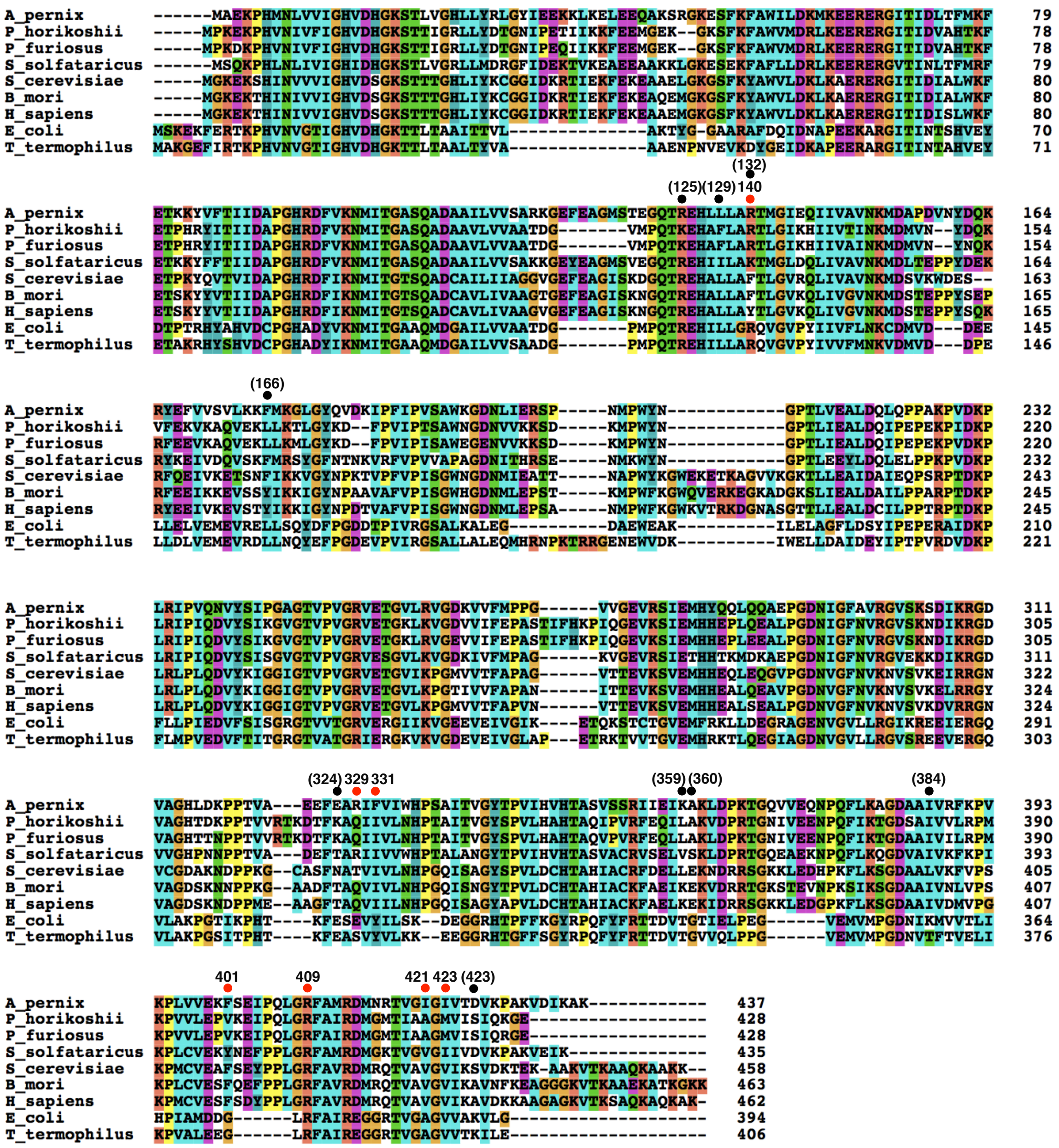

Figure S6. Amino acids sequence alignment of EF1A (EF-Tu). The sequences in archaea, *A. pernix* (A_pernix), *P. horikoshii* (P_horikoshii), *P. furiosus* (P_furiosus), and *S. solfataricus* (S_solfataricus), in eukaryotes, *S. cerevisiae* (S_cerevisiae), *B. mori* (B_mori), and *H. sapiens* (H_sapiens), and in bacteria, *Escherichia coli* (E_coli), and *Thermus thermophiles* (T_termophilus), were aligned using the program Clustal W (http://www.genome.jp/tools-bin/clustalw). Clustal X (http://www.clustal.org/clustal2/) was used to prepare the figure. The red dots above the sequences indicate the residues that participate in the interaction with $_{Ape}$P1 in the presence of GTP and $_{Ape}$Pelota (present study), while the black dots indicate the positions bound to $_{Pho}$P1 in the presence of GDP (Ito et al., 2014).

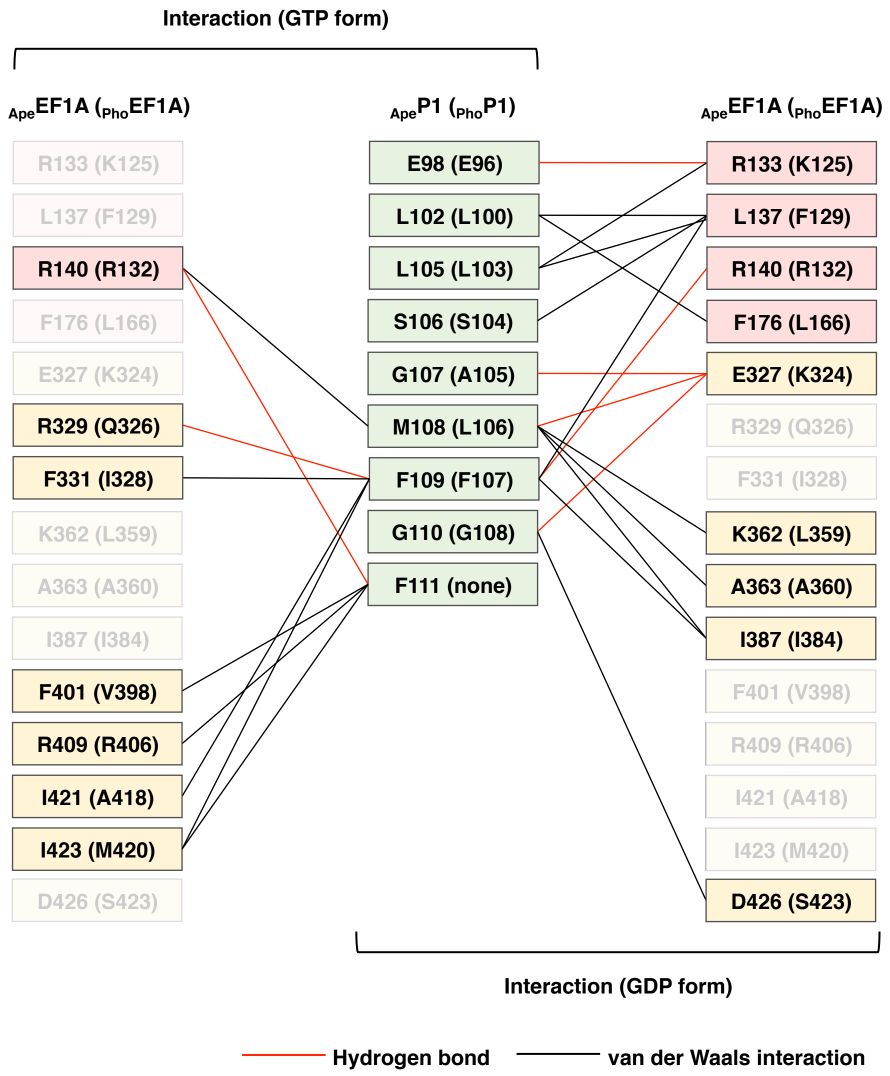

Figure S7. Schematic diagram of the switching of the aP1 interactions between GTP- and GDP-bound forms of aEF1A. Van der Waals contacts and hydrogen bonds are represented by black and red lines, respectively. The amino acid numbers are for *Aeropyrum pernix* proteins, and the numbers in parentheses are *Pyrococcus horikoshii* proteins.

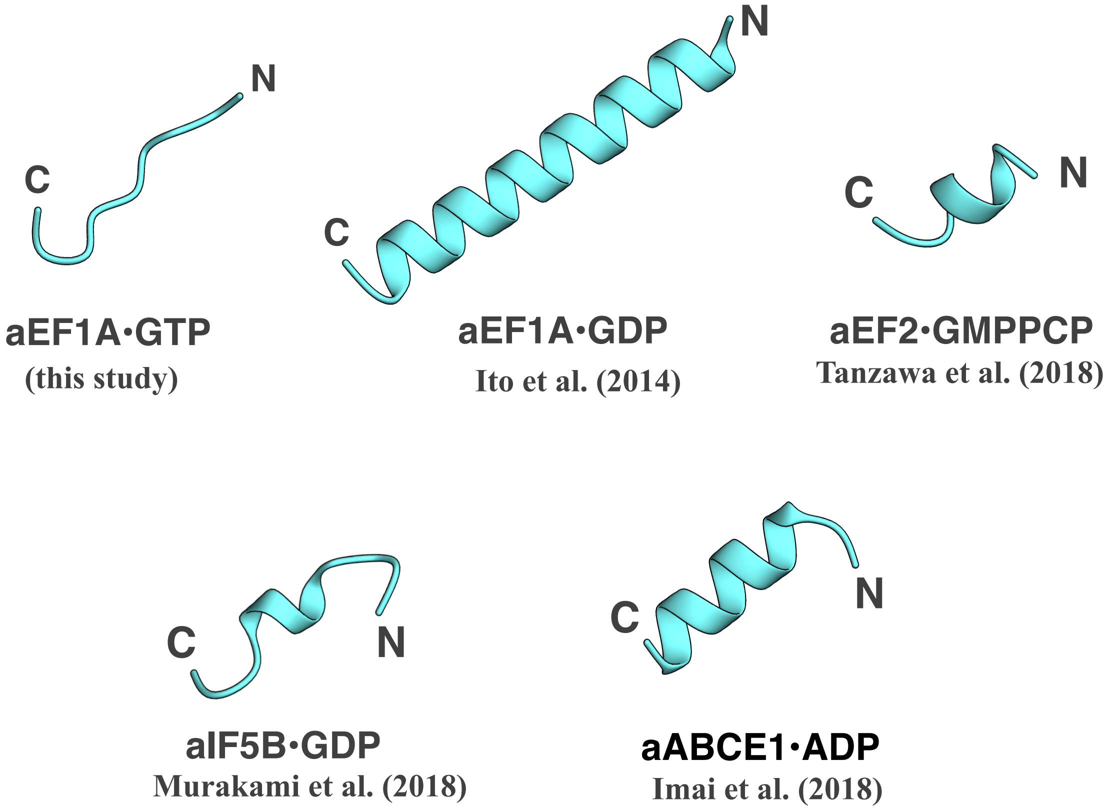

Figure S8. Comparison of the structures of the C-terminal region in aP1 bound with various translation factors. Crystal structures of the C-terminal regions of aP1 bound to $_{Ape}$EF1A•GTP•$_{Ape}$Pelota (A) [PDB ID: 3WXM], $_{Pho}$EF1A•GDP (B) [PDB ID: 3WY9], $_{Pho}$EF2•GMPPCP (C) [PDB ID: 5H7L], $_{Ape}$IF5B•GDP (D) [PDB ID: 5YT0], and $_{Pfu}$ABCE1•ADP (E) [PDB ID: 5YV5].

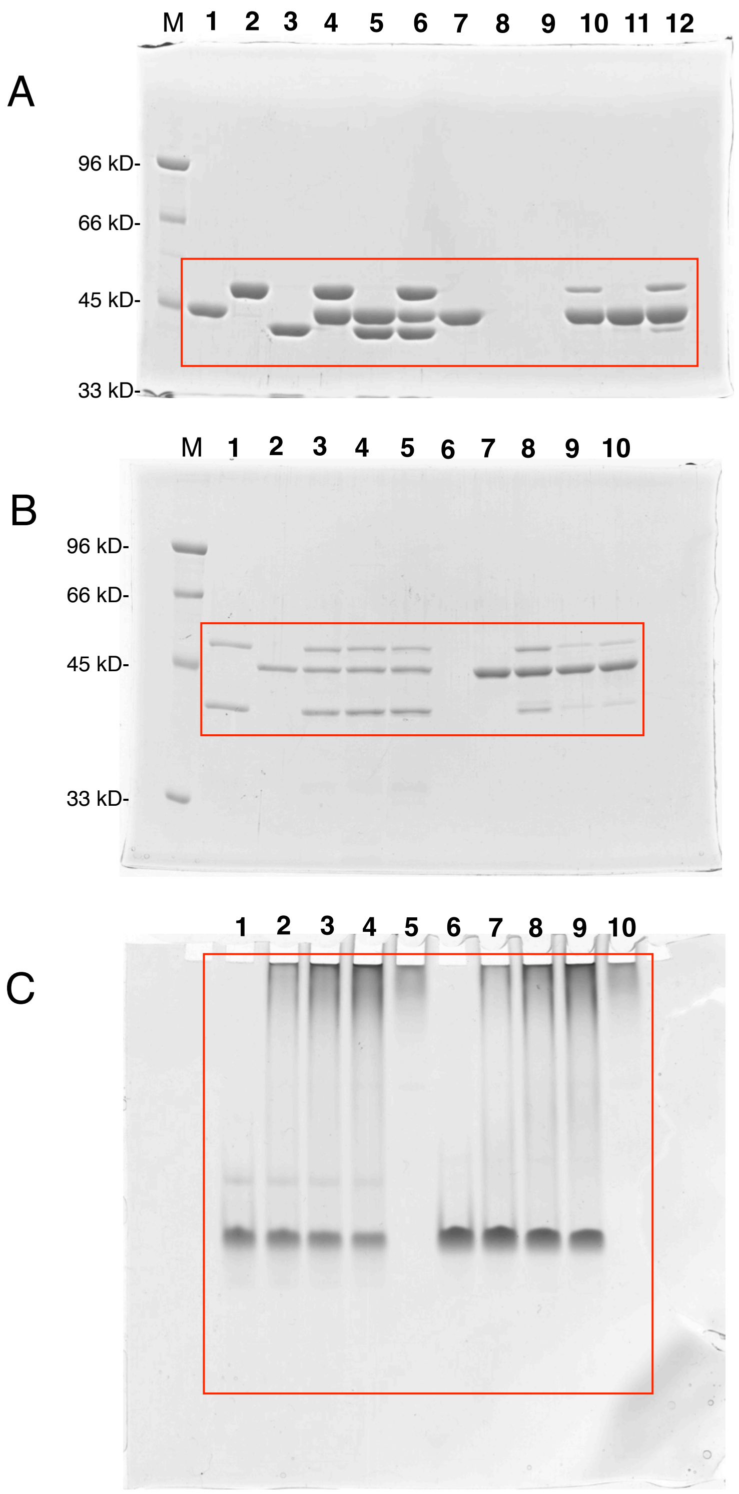

Figure S9. Original gel images used in Figures 1A (A), 3 (B), and S5 (C). A and B, SDS-PAGE patterns in the pull-down assay. Samples for lanes 1 to 12 in A, described in the legend for Figure 1A, and samples for lanes 1 to 10 in B, described in the legend for Figure 3 were subjected to SDS-PAGE together with protein size marker (M) purchased from Bio-Rad® (cat. # 161-0304). C, Native-PAGE pattern in the binding assay. Samples for lanes 1 to 10 are described in the legend for Figure S5. After electrophoresis, the gels were stained with Coomassie Brilliant Blue from Wako® (cat. # 038-17932). Using Epson scanner ES-2200, the stained gels were digitalized by scanning and saving at 300 dpi. Red boxes in gels A, B and C show the regions cropped for Figures 1A, 3, and S5, respectively.

Supplementary Tables

Table S1. Data collection and refinement statistics.

	**aP1-C17•aEF1A•GTP•aPelota**
**Data collection**	
Space group	*P*1
Unit-cell parameters (Å, º)	a = 71.45, b = 73.41, c = 108.14
	(α = 98.64, β = 93.80, γ = 100.44)
Resolution range (Å)	50.0–3.00 (3.05–3.00)[a]
No. of measured reflections	164457
No. of unique reflections	41897
Completeness (%)	98.9 (99.0)
Redundancy	3.9 (3.9)
Average I/σ(I)	20.4 (2.4)
R_{merge}[b] (%)	7.0 (56.4)

[a] Values in parentheses are for the highest resolution shell.

[b] $R_{merge} = \sum_{hkl}\sum_i |I_i(hkl) - \langle I(hkl)\rangle| / \sum_{hkl}\sum_i I_i(hkl)$, where $I_i(hkl)$ is the *i*-th intensity measurement of reflection *hkl*, including symmetry-related reflections, and $\langle I(hkl)\rangle$ is its average.

Table S2. Refinement statistics.

	aP1-C17•aEF1A•GTP•aPelota
R_{work} / R_{free}^{a} (%)	19.9/ 27.9
No. of complex	2
**No. of atoms**	
Protein	12380
Ligand	64
Solvent	104
**Average *B*-factors (Å^2)**	
Protein	82.7
Ligand	79.1
Solvent	55.5
**RMS deviations**	
Bond lengths (Å)	0.007
Bond angles (º)	1.316
**Ramachandran plot**	
Favored region (%)	94.99
Allowed region (%)	4.06
Outlier region (%)	0.95

[a] R_{free} was calculated by using 5% of randomly selected reflections that were excluded from the refinement.

Table S3. Effect of mutations in $_{Pho}$EF1A on aP1 binding.

aEF1A	K_d (μM)*
WT	23.6 ± 1.3
R132(R140)A	87.8 ± 12.0
Q326(R329)A	11.1 ± 0.8
I328(F331)S	93.3 ± 10.2
V398(F401)S	38.3 ± 2.8
R406(R409)A	33.0 ± 2.1
M420(I423)S	80.1 ± 8.7
I328(F331)S/M420(I423)S	n.d.

The FITC-labeled peptide of $_{Pho}$P1C14 was mixed with various concentrations of the complex composed of each $_{Pho}$EF1A mutant, GTP, and $_{Pfu}$Pelota, and mP values were determined for individual concentrations of the complex. Binding data were fitted to a nonlinear regression using GraphPad Prism 6. K_d values represent the average of three replicate experiments.

Table S4. Primers used in this study.

Name	Sequence	Notes
1	TGGTGCCGCGCGGCAGCCATATGGCTGAGAAGCCGCATATGA	Forward cloning primer for *A. pernix* aEF1A
2	GTTAGCAGCCGGATCCTCGAGCCAGCTTGGCCTTTATGTCTACC	Reverse cloning primer for *A. pernix* aEF1A
3	GAGAGACATATGGAGATATTGGAAGAAAAACC	Forward cloning primer for *P. furiosus* aPelota
4	GAGAGAGGATCCTTACTTGATTTTAAACCTCAACAG	Reverse cloning primer for *P. furiosus* aPelota
5	GAGAGACATATGCGCGTCGAGGTCCTCGACAATAAAAGGAGG	Forward cloning primer for *A. pernix* aPelota
6	GAGAGAAAGCTTTTATAGCCTCCTAGCCTCCTGCG	Reverse cloning primer for *A. pernix* aPelota
7	GGGAAGGATTTCAGAATTCTGTGAAGAGGAAGCCCTTGC	Forward cloning primer for *P. horikoshii* MBP-aP1C14
8	GCAAGGGCTTCCTCTTCACAGAATTCTGAAATCCTTCCC	Reverse cloning primer for *P. horikoshii* MBP-aP1C14
9	GCCTTCCTTGCAGCGACGCTCGGTATCAAG	Forward mutagenesis primer for *P. horikoshii* aEF1A-R132A
10	CTTGATACCGAGCGTCGCTGCAAGGAAGGC	Reverse mutagenesis primer for *P. horikoshii* aEF1A-R132A
11	GATACATTCAAGGCCGCGATCATAGTCCTTAAC	Forward mutagenesis primer for *P. horikoshii* aEF1A-Q326A
12	GTTAAGGACTATGATCGCGGCCTTGAATGTATC	Reverse mutagenesis primer for *P. horikoshii* aEF1A-Q326A

13	CAAGGCCCAGATCAGCGTCCTTAACCACCC	Forward mutagenesis primer for *P. horikoshii* aEF1A-I328S
14	GGGTGGTTAAGGACGCTGATCTGGGCCTTG	Reverse mutagenesis primer for *P. horikoshii* aEF1A-I328S
15	GTAGTCCTTGAGCCCAGCAAGGAGATTCCAC	Forward mutagenesis primer for *P. horikoshii* aEF1A-V398S
16	GTGGAATCTCCTTGCTGGGCTCAAGGACTAC	Reverse mutagenesis primer for *P. horikoshii* aEF1A-V398S
17	GATTCCACAGCTCGGTGCGTTTGCCATTAGAG	Forward mutagenesis primer for *P. horikoshii* aEF1A-R406A
18	CTCTAATGGCAAACGCACCGAGCTGTGGAATC	Reverse mutagenesis primer for *P. horikoshii* aEF1A-R406A
19	CAATAGCTGCTGGTAGCGTAATATCTATTC	Forward mutagenesis primer for *P. horikoshii* aEF1A-M420S
20	GAATAGATATTACGCTACCAGCAGCTATTG	Reverse mutagenesis primer for *P. horikoshii* aEF1A-M420S
